# Massive Survey on Bacterial–Bacteriophages Biodiversity and Quality of Natural Whey Starter Cultures in Trentingrana Cheese Production

**DOI:** 10.3389/fmicb.2021.678012

**Published:** 2021-06-14

**Authors:** Andrea Mancini, Maria Cid Rodriguez, Miriam Zago, Nicola Cologna, Andrea Goss, Ilaria Carafa, Kieran Tuohy, Andrea Merz, Elena Franciosi

**Affiliations:** ^1^Food Quality and Nutrition Department, Research and Innovation Centre, Fondazione Edmund Mach (FEM), San Michele all’Adige, Italy; ^2^Centro di ricerca Zootecnia e Acquacoltura (CREA-ZA), Lodi, Italy; ^3^Trentingrana Consorzio dei Caseifici Sociali Trentini s.c.a., Trento, Italy

**Keywords:** bacteriophages, natural whey starter cultures, *Lactobacillus helveticus*, Grana-like cheese, *Levilactobacillus brevis*

## Abstract

This study focused on the microbial and bacteriophages identification and characterization in cheese-production facilities that use natural whey starter (NWS) cultures for Trentingrana production. Bacterial and phage screening was carried out on cooked not acidified whey and NWS samples isolated from six dairy factories, for 4 consecutive days in four different months. By means of a combined approach, using plate counts, bacterial isolation, and metataxonomic analysis *Lactobacillus helveticus* was found occurring as the dominant species in NWS cultures and *Levilactobacillus brevis* as codominant in the cheese factories where the temperature of NWS production was mainly lower than 40°C, suggesting that the variability in the parameters of the NWS culture preparation could differently modulate the bacterial species in NWS cultures. Using turbidity test approach on 303 bacterial isolates from the NWS cultures, 120 distinct phages were identified. *L. helveticus* phage contamination of NWS cultures was revealed in most of the analyzed samples, but despite the great recovery of bacteriophage contamination cases, the microbial quality of NWS cultures was high. Our results support the presence of natural bacteriophage resistance mechanisms in *L. helveticus*. The use of NWS cultures probably creates an ideal environment for the proliferation of different *L. helveticus* strains balanced with their phages without a clear dominance. It is evident, from this study, that the presence of a high biodiversity of NWS bacterial strains is relevant to avoid phages dominance in NWS cultures and consequently to keep a good acidification ability.

## Introduction

Traditional and artisanal cheese productions are often based on fermentation processes carried on by defined starter cultures used to achieve defined and typical flavors and/or textures (Gatti et al., 2014; Gobbetti et al., 2018b). Natural whey starters (NWSs) are traditionally used in Italian long ripened hard cheese production as for Parmigiano Reggiano, Grana Padano, and Trentingrana (Bertani et al., 2020). Refreshed daily in the dairy factory from whey collected at the end of the cheese-making process (Bertani et al., 2020), these starters are mainly characterized by thermophilic lactic acid bacteria (LABs) such as *Lactobacillus helveticus*, *Lactobacillus delbrueckii* ssp. *bulgaricus* and *L. delbrueckii* ssp. *lactis* (Rossetti et al., 2008).

Together with raw milk quality, bacteriophages (or phages) may represent a dealing factor able to negatively affect fitness and performance of dairy starter cultures (Carminati et al., 2011). Phages are viruses able to infect bacteria; they are present in all the environments, including niches related to human activities as in the case of dairy factories (Carminati et al., 2011). Despite intense efforts as adapted factory design, sanitations, adequate ventilation, and culture rotation, a complete phage eradication in the dairy industry remains a utopian goal, and phage infection of starter LABs is still the most common cause of slow and/or incomplete fermentation (Guglielmotti et al., 2012). Consequently, high pH values and residual lactose may promote growth of pathogenic or spoilage bacteria, negatively affecting the quality or the yield of the final product (Josephsen and Neve, 1998). The most permanent source of phage in dairy is the raw milk, and considering that Trentingrana dairy factories use raw milk in cheese production, they can rapidly grow up to high concentration in NWS cultures (Guglielmotti et al., 2012).

Previous works showed the coexistence in NWS for Grana Padano of phage ecologically related to bacterial strains belonging to species such as *L. helveticus* and *L. delbrueckii* (Zago et al., 2006, 2008). As in other ecosystems, bacteriophages play an ecological role within NWS culture, acting as a biological pressure agent following the “kill-the-winner” hypothesis. NWS culture bacteriophages lead the natural selection of phage-resistant bacterial strains, where the fastest-growing bacteria are inhibited in dominating the NWS community, thus preserving the overall technological performances of the NWS cultures (Zago et al., 2008; Gobbetti et al., 2018a). Therefore, the comprehension of bacteria and phage biodiversity dynamic in NWS cultures is of prominent importance, and a good management of NWS processing for the Grana-like cheese production should consider the overall players in this ecological niche.

It has been demonstrated that small changes in the technological parameters, such as curd cooking temperature, titratable acidity, and pH, could affect the bacterial consortium present in NWS cultures (Rossetti et al., 2008). Despite the findings provided by these studies, the microbial and bacteriophage biodiversity of NWS culture is not fully known. To the best of our knowledge, this is the first study reporting the dynamics of bacteria and phage biodiversity in NWS by means of a massive sampling system in cheese factories following different NWS technologies of production. Therefore, in this study, we focused on NWS, collected just before addition to the vat milk, and cooked not acidified whey (cNAW), collected at the end of curdle cooking.

The samples were collected from six selected Trentingrana dairies, over 1 year of production, for 144 samples: 72 cNAW and 72 NWS samples.

This study was performed to understand the dynamics of bacteria and phages of NWS cultures over time, so in this work, we (i) characterized the dynamic of bacterial and phage community of the NWS collected to increase the existing knowledge and (ii) investigated if and how the cheese factory technology of NWS production affects the bacterial and phage community in the NWS cultures. The final goal is to maintain the high quality of Trentingrana production, avoiding economic loss associated with failure in milk fermentation and low cheese yield.

## Materials and Methods

### Collection of cNAW and NWS Samples, and Determination of Temperatures and pH

cNAW and NWS culture samples used in Trentingrana manufacturing were collected for four consecutive days in four different months (February, May, August, and November 2018) from six dairy factories (A–F) located in the province of Trento (north eastern of Italy) and operating into the Trentingrana Protected Designation of Origin cheese area of production (total of 144 samples).

The Trentingrana production had stopped at cheese factory C for July and August months, so NWS and cNAW samples were collected in September only for cheese factory C.

NWS titratable acidity was measured and expressed as Soxhlet–Henkel degrees (°SH); the pH was determined using a pH electrode (Crison Instruments, Barcelona, Spain). Samples were shipped to the laboratory under liquid nitrogen and stored at −80°C before the analysis.

The temperature of the room and of the NWS tank has been determined using Testo 175 T2 (Testo Ltd., Alton, United Kingdom). Temperature was recorded each minute for the overnight fermentation of the NWS.

### Microbiological Counts and Isolation

All the samples were diluted in sterile peptone water and plated onto whey agar medium (WAM) according to Gatti et al. (2003) for cultivating thermophilic lactobacilli in anaerobic conditions for 72 h at 45°C. All culture media and anaerobic system were purchased from Oxoid (ThermoFisher Scientific, Milan, Italy).

Eight colonies grown onto WAM plates were randomly isolated from NWS samples (plates with a number of colonies in the range of 10–300). Each isolate was purified by subsequent culturing in whey broth (the broth version of the WAM used for plate counting). Pure cultures were stored at −80°C in glycerol (40% vol/vol) stocks. Cell morphology was determined by microscopic observation, Gram characterization was performed applying the KOH method (Gregersen, 1978), and catalase activity was tested by adding 5% H_2_O_2_ drops on the colonies.

### DNA Extraction and Genotypic Identification of the NWS Bacteria

All bacterial isolates were grown 48 h in the whey broth culture at 45°C before DNA extraction. The bacterial DNA was isolated using Quick-gDNA^TM^ MicroPrep (Zymo Research, Italy) following the manufacturer’s instructions.

Randomly amplified polymorphic DNA–polymerase chain reaction (RAPD-PCR) was carried out in a total volume of 25 μL using primer M13 (Rossetti and Giraffa, 2005). Cluster analysis of DNA patterns was carried out using GelCompar II-BioNumerics software (package version 6.0; Applied Maths, bioMérieux, Belgium), exploiting the unweighted pair group method arithmetic averages. Similarity of PCR fingerprint profiles was calculated based on Pearson product–moment correlation coefficient. The threshold breakpoint value was fixed to 80%; isolates with similarity coefficient higher than 80% were classified into the same cluster, according to Gatti et al. (2003).

One isolate representative of each biotype was genotypically identified by 16S rRNA gene analysis. A fragment of the 16S rRNA gene was amplified using the primers 27F (5′-GAGAGTTTGATCCTGGCTCAG-3′) and 1495R (5′-CTACGGCTACCTTGTTACGA-3′), designed by Grifoni et al. (1995). The PCR products were purified using the Exo-SAP-IT^TM^ kit (USB Co., Cleveland, OH, United States) and sequenced in an ABI PRISM 3100 sequencer (Applied Biosystems, Foster City, CA, United States), using the BigDye Terminator v1.1 cycle sequencing kit (Applied Biosystems). The obtained sequences were compared using the BLAST algorithm available on the National Center for Biotechnology Information (NCBI, United States). All amplifications were run in a T100^TM^ ThermalCycler (Bio-Rad Laboratories, Hercules, CA, United States).

### DNA Extraction, MiSeq Library Preparation, and Illumina Sequencing

Illumina analysis was performed on 24 cNAW and 24 NWS samples (one cNAW and NWS sample for each month and cheese factory). Three milliliters of sample was centrifuged at 3,200 × *g* for 15 min at 4°C. The genomic DNA was extracted from the pellet using the Power Food^TM^ Microbial DNA Isolation Kit (Qiagen, Hilden, Germany) according to the manufacturer’s instructions. All DNA samples were purified by PowerClean DNA Clean-up Kit (Qiagen). The DNA quality and concentrations were determined by NanoDrop^TM^ 8000 Microvolume UV-Vis spectrophotometer (ThermoFisher Scientific).

Amplicon library preparation, quality and quantification of pooled libraries, and pair-end sequencing using the Illumina MiSeq system (Illumina, United States) were carried out at the Sequencing Platform in Fondazione Edmund Mach (FEM, San Michele all’Adige, Italy). Briefly, for each sample, a 464-nucleotide sequence of the V3–V4 region (Baker et al., 2003; Claesson et al., 2010), of the 16S rRNA gene (*Escherichia coli* positions 341 to 805) was amplified. Unique barcodes were attached before the forward primers to facilitate the pooling and subsequent differentiation of samples. To prevent preferential sequencing of the smaller amplicons, the amplicons were cleaned using the Agencourt AMPure kit (Beckman Coulter, Brea, CA, United States) according to the manufacturer’s instructions; subsequently, DNA concentrations of the amplicons were determined using the Quant-iT PicoGreen dsDNA kit (Invitrogen, ThermoFisher Scientific) following the manufacturer’s instructions. In order to ensure the absence of primer dimers and to assay the purity, the generated amplicon libraries quality was evaluated by a Bioanalyzer 2100 (Agilent, Palo Alto, CA, United States) using the High Sensitivity DNA Kit (Agilent). Following the quantitation, cleaned amplicons were mixed and combined in equimolar ratios.

### Illumina Data Analysis and Sequences Identification by QIIME2

Raw paired-end FASTQ files were demultiplexed using idemp^1^ and imported into Quantitative Insights Into Microbial Ecology (Qiime2, version 2020.8). Sequences were quality filtered, trimmed, denoised, and merged using DADA2 (Callahan et al., 2016). Chimeric sequences had been identified and removed *via* the consensus method in DADA2. Representative bacterial sequences had been aligned with MAFFT and used for phylogenetic reconstruction in FastTree using plugins alignment and phylogeny (K. Price et al., 2009; Katoh and Standley, 2013). *α*- and *β*-diversity metrics had been calculated using the core-diversity plugin within QIIME2 and visualized by emperor (Vazquez-Baeza et al., 2013). Bacterial taxonomic and compositional analyses were carried on by using plugins feature-classifier^2^. A pretrained naive Bayes classifier based on the Greengenes 13_8 99% operational taxonomic unit (OTU) database, which had been previously trimmed to the V4 region of 16S rDNA, bound by the 341F/805R primer pair, was applied to paired-end sequence reads to generate taxonomy tables. The data generated by MiSeq Illumina sequencing were deposited in the NCBI Sequence Read Archive and are available under Ac. Number PRJNA695135^3^ from sample SAMN17602423 to SAMN17602468.

### Enrichment, Isolation, and Purification of Phages

All the 72 samples of NWS cultures were screened for the presence of bacteriophages. The related cNAW samples were used to isolate the virulent phages, which used the NWS isolated bacteria as host. Phage enrichment was performed as previously reported by Zago et al. (2005). Briefly 1 g of CaCO_3_ was included in tubes of 40-mL cNAW sample, acidified with 0.5 mL of sodium maleate buffer (maleic acid 0.05 M titrated with 0.2 N NaOH until pH 5.15 is reached), and then inoculated with 1 mL of NWS from the same sampling day and dairy. After anaerobic incubation at 45°C for 72 h, the tubes were centrifuged at 4,000 revolutions/min for 15 min at 4°C. Supernatants obtained from the enrichment step were filtered with 45-μm membrane filter pore size (Merck–Millipore, Darmstadt, Germany) and checked for the presence of phages by means of turbidity test.

Tubes of WAM-Ca broth (WAM supplemented with 10 mM CaCl_2_) were added with 5% of the previously filtered supernatants and inoculated with 2% mL of host culture in the logarithmic growth phase. The tubes were incubated at 45°C and observed each 6 h until cell lysis occurred. The lysis was detected by the higher clearness of the broth, compared to controls (tubes without addition of the supernatants), which were more turbid due to bacteria growth. After 24 h, where no lysis was detected, the bacterial cultures were used for 2% inoculation of new tubes of WAM-Ca broth. After four rounds of inoculations without lysis, the bacterial isolate was considered as not suitable for the phage isolation. Where lysis was observed, the tubes were then filtered through 0.45-μm membrane filter pore size (Merck–Millipore), set to pH 7, and stored at 4°C until use, for no more than 7 days. Phage stocks were stored at −80°C with 40% vol/vol of glycerol.

### Titer Determination of Phage

Phage titer was expressed as the number of plaque-forming units per mL of sample (pfu/mL). The suspension of the phage was diluted sevenfold by using phosphate-buffered saline added with CaCl_2_ 10 mM. One hundred thirty microliters of the host bacteria in the logarithmic growth phase was added to 5 mL of semisolid WAM-Ca (WAM-Ca, 4% wt/vol agar). The mixture was poured on the surface of WAM-Ca plates and then inoculated with 10 μL of phage dilutions in seven plate sectors. After anaerobic incubation at 45°C, clear plaques were checked. The phage titer was calculated using the formula titer (pfu/mL) = plaque number × dilution factor × 100.

### Detection of the Lysogenic State

Each isolated bacterial biotype, characterized as a phage host, was tested for the detection of prophage, adapting the method of Cochran and Paul (1998). Overnight WAM cell cultures were split into two aliquots: the first aliquot was added with mitomycin C (MytC^+^, 1 μg/mL); the second aliquot was not added with MytC^+^ and used as control. After 24 h of incubation at room temperature, in the dark, induced phages were detected as follows: 100 μL of each cell suspension was used to inoculate 7 mL of soft WAM (WAM, 0.7% wt/vol agar) plated on a layer of 1.8% (wt/vol) agar and incubated for 24 h at the optimal conditions for each strain. The growth reduction onto MytC^+^ plates, after comparison with control plates, indicated the presence of phage.

If not specified, all chemical compounds were purchased from Sigma–Aldrich (St. Louis, MO, United States).

### Statistical Analysis

A normality test (Shapiro-Wilk *W*) was performed, as well as a nonparametric test (Kruskal--Wallis) analyzing the day of collection as the independent variable and the microbial plate counts as the dependent variable. All the tests on plate counts were performed using the STATISTICA data analysis software system, version 9.1 (StatSoft, Inc, 2010^4^).

Differences in diversity indices (OTUs number and Shannon diversity index) of different samples were tested by Kruskal–Wallis test by a plug-in implemented in QIIME2. The overall structural changes of bacterial community were visualized by principal coordinate analysis (PCoA) based on both weighted and unweighted UniFrac distance matrices. The statistical significance was assessed *via* the nonparametric PERMANOVA (permutational multivariate analysis of variance) by means of plug-in implemented in QIIME2. For the differential abundance test, the taxonomy information for each OTU sequence was provided using ANCOM method (Mandal et al., 2015) by means of plug-in implemented in QIIME2.

## Results

### Thermophilic Bacteria Counts in Trentingrana NWS and cNAW Samples and Technological Process of NWS Production

The WAM counts of thermophilic bacteria in NWS and cNAW samples during the monitoring from February to December are shown in Table 1. NWS thermophiles varied without significant differences in the same and among the different dairy factories, ranging between 7.3 and 8.8 log colony-forming units (cfu)/mL. Only in November, at dairy factory D, NWS cultures’ averaged value was lower than 7.3 (6.7 log cfu/mL). The dairy factory C was the only one showing significant differences in the NWS thermophilic counts that were lower in February (7.5 log cfu/mL) and higher in August (8.6 log cfu/mL). More variability was observed in the cNAW thermophilic counts that ranged between 4.8 and 7.1 log cfu/mL. With the exceptions of dairy factories B and F, cNAW thermophilic counts were showing significant differences during the year without a well-defined trend.

**TABLE 1:** Thermophilic lactic acid bacteria (TLAB) counts onto WAM of Trentingrana NWS and cNAW.

	**A**	**B**	**C**	**D**	**E**	**F**	**TOT (*n* = 18)**
**NWS TLAB**							
February	7.4 ± 0.39	7.8 ± 0.35	7.5 ± 0.38^*A*^	7.3 ± 0.39	7.3 ± 0.63	7.6 ± 0.31	7.5 ± 0.41
May	7.7 ± 0.29	8.0 ± 1.13	7.9 ± 0.35^*AB*^	7.6 ± 0.39	7.8 ± 0.30	8.0 ± 0.77	7.8 ± 0.50
August	8.3 ± 0.57	8.3 ± 0.84	*8.6 ± 0.33^*B*^	7.3 ± 0.98	8.1 ± 0.61	8.7 ± 0.48	8.2 ± 0.76
November	7.6 ± 0.82	8.8 ± 0.60	8.3 ± 0.81^*AB*^	6.7 ± 0.44	7.4 ± 0.57	8.2 ± 0.59	7.8 ± 0.90
TOT (*n* = 12)	7.8 ± 0.73	8.3 ± 0.74	8.0 ± 0.61	7.2 ± 0.65	7.7 ± 0.61	8.1 ± 0.62	
**cNAW TLAB**							
February	5.3 ± 0.30^*A*^	6.4 ± 0.50	7.1 ± 0.07^*C*^	4.8 ± 0.01^*A*^	6.5 ± 0.73^*B*^	5.7 ± 0.25	6.0 ± 0.92
May	5.5 ± 0.14^*A*^	6.1 ± 0.84	5.3 ± 0.41^*A*^	5.8 ± 0.71^*B*^	4.9 ± 0.14^*A*^	6.2 ± 0.98	5.7 ± 0.65
August	6.8 ± 0.54^*B*^	6.2 ± 0.07	*5.8 ± 1.4^*AB*^	6.4 ± 0.64^*B*^	6.1 ± 0.06^*B*^	5.8 ± 0.10	6.2 ± 0.31
November	6.6 ± 0.09^*B*^	6.7 ± 0.47	6.4 ± 0.26^*BC*^	5.7 ± 0.47^*AB*^	6.5 ± 0.43^*B*^	6.9 ± 0.17	6.5 ± 0.47
TOT (*n* = 12)	6.1 ± 0.68	6.4 ± 0.49	6.2 ± 0.77	5.6 ± 0.75	6.2 ± 0.63	6.2 ± 0.62	

*All the samples were collected from six dairy factories labeled from A to F, four times a year, from February to November 2018. The plate count data were expressed as means ± standard deviation of bacterial number of cfu/mL transformed to log10.*

For a given column and dairy of sampling, microbial count, pH, and temperature values with A, B, and C superscripts are significantly different (*P* < 0.05).

*At cheese factory C, the samples were collected at the end of September because in August the Trentingrana production stopped.

The NWS pH mean values ranged between 3.3 and 3.6 and the titratable acidity between 28°SH and 32°SH / 50 mL without any significant difference for month or cheese factory (data not shown).

As the final acidification activity in NWS cultures is directly associated with a slow temperature decrease from 55°C to 40°C during the overnight process of NWS production (Di Cagno et al., 2006), the NWS production cycle was split into two stages: a first thermophilic stage, when the cNAW is cooled from cooking temperature (55°C) to 40°C, and a second mesophilic stage, after the first cNAW cooling, when the temperature was in the range between 20°C and 40°C (Table 2).

**TABLE 2:** Length in minutes of the first thermophilic and second mesophilic stage of the overnight NWS culture production cycle.

	**A**	**B**	**C**	**D**	**E**	**F**
**First thermophilic stage (T° over 40°C)**						
February	537 ± 58^*A*^	372 ± 27^*A*^	447 ± 5^*AB*^	722 ± 74^*A*^	598 ± 9^*A*^	595 ± 32^*A*^
May	549 ± 53^*A*^	344 ± 34^*A*^	525 ± 45^*B*^	705 ± 52^*A*^	635 ± 43^*AB*^	782 ± 14^*C*^
August	555 ± 67^*A*^	552 ± 45^*B*^	*388 ± 36^*A*^	739 ± 6^*A*^	629 ± 74^*AB*^	683 ± 25^*B*^
November	512 ± 62^*A*^	510 ± 38^*B*^	438 ± 14^*AB*^	773 ± 63^*A*^	652 ± 37^*B*^	519 ± 49^*A*^
TOT (*n* = 12)	538 ± 59	444 ± 42	560 ± 24	735 ± 62	629 ± 59	645 ± 37
**Second mesophilic stage (T° between 20 and 40°C)**						
February	757 ± 58^*B*^	1,020 ± 48^*D*^	891 ± 12^*C*^	318 ± 74^*B*^	644 ± 5^*AB*^	705 ± 3^*BC*^
May	744 ± 53^*B*^	1,098 ± 44^*D*^	801 ± 45^*C*^	319 ± 52^*B*^	565 ± 15^*A*^	610 ± 24^*A*^
August	721 ± 67^*B*^	804 ± 59^*C*^	*893 ± 37^*C*^	361 ± 65^*B*^	578 ± 74^*A*^	670 ± 25^*B*^
November	763 ± 62^*B*^	786 ± 52^*C*^	843 ± 14^*C*^	327 ± 63^*B*^	655 ± 37^*B*^	751 ± 49^*C*^
TOT (*n* = 12)	746 ± 61	927 ± 52	857 ± 38	321 ± 64	611 ± 39	684 ± 37

*All the times were recorded from six dairy factories, labeled from A to F, four times a year, from February to November 2018.*

For a given column and dairy of sampling, the values with A, B, and C superscripts are significantly different (*P* < 0.05).

*At cheese factory C, the samples were collected at the end of September because in August the Trentingrana production stopped.

At dairy plants A, B, and C, the first thermophilic was at least 2 h 25 min shorter than the second mesophilic stage, conversely at dairy plant D, the first was approximately 7 h longer than the second stage. At cheese factories E and F, there was no relevant difference between the first and second stage of NWS culture production cycle.

### Identification of Bacteria From NWS Cultures

A total of 576 isolates were picked up from WAM agar plates. A RAPD-PCR fingerprinting had been performed (i) to preliminarily recognize the bacterial isolates purified from colonies onto the same plate originated from genetically alike single mother cells and (ii) to investigate the microbial diversity beyond the species level, establishing the number of different biotypes inside cheese factory and over time. One isolate for each biotype was selected for further identification by 16S rRNA gene sequencing.

**FIGURE 1:**
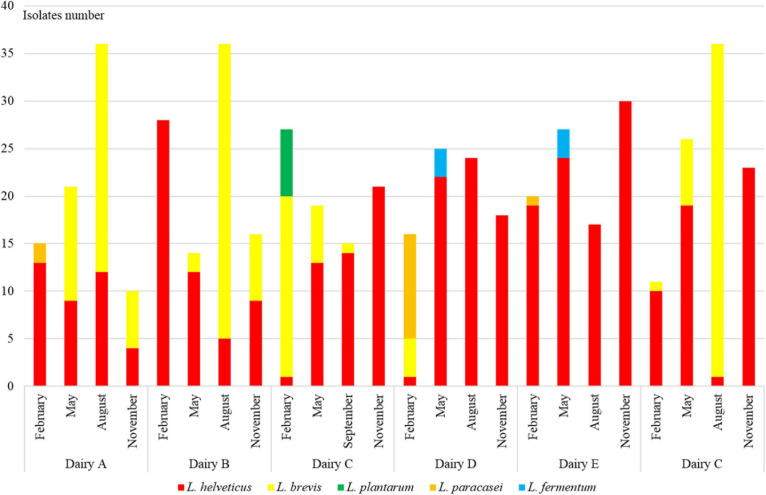
Distribution of *L. helveticus*, *L. brevis*, *L. paracasei*, *L. plantarum*, and *L. fermentum* isolates in NWS samples collected from six dairy factories labeled from A to F four times in 2018 year: February, May, August, and November. At cheese factory C, the samples were collected at the end of September because in August the Trentingrana production stopped.

Of the 576 isolates, 45 did not grow after plate isolation and were discarded, 383 had been identified as *L. helveticus*, 120 as *Levilactobacillus brevis*, 13 as *Lacticaseibacillus paracasei*, eight as *Lactiplantibacillus plantarum*, and the other seven as *Limosilactobacillus fermentum* (Figure 1).

Cluster analysis of RAPD-PCR profiles showed that, at a similarity level of 85%, *L. helveticus* and *L. brevis*, accounting for the 95% of all the isolates, grouped, respectively, into 243 and 51 different biotypes. *L. paracasei*, *L. plantarum*, and *L. fermentum* grouped into two, three, and five different biotypes, respectively (Table 3).

**TABLE 3:** Distribution of *L. helveticus*, *L. brevis*, *L. paracasei*, *L. plantarum*, and *L. fermentum* biotypes (n° BioT) in NWS samples collected from six dairy factories labeled from A to F four times a year from February to November 2018.

	**A**	**B**	**C**	**D**	**E**	**F**	**TOT n° BioT**
***L. helveticus***							
February n° BioT	8	23	1	1	12	5	50
May n° BioT	8	11	10	17	18	11	73
August n° BioT	7	4	*8	14	11	16	60
November n° BioT	4	4	14	14	11	15	61
TOT n° BioT	27	42	33	46	52	47	243
Biodiversity (%)	0.71	0.78	0.67	0.72	0.58	0.54	0.63
***L. brevis***							
February n° BioT	—	—	14	3	—	1	18
May n° BioT	7	1	4	—	—	2	14
August n° BioT	7	8	*1	—	—	—	16
November n° BioT	1	2	—	—	—	—	3
TOT n° BioT	15	11	19	3	—	3	51
Biodiversity (%)	0.36	0.28	0.73	0.75	—	0.38	0.43
***L. paracasei***							
February n° BioT	1			1			2
Biodiversity (%)	0.50	—	—	0.09	—	—	0.15
***L. plantarum***							
February n° BioT			2		1		3
Biodiversity (%)	—	—	0.29	—	1	—	0.38
***L. fermentum***							
May n° BioT	—	—	—	2	2	1	5
Biodiversity (%)	—	—	—	0.67	0.67	1	0.71
TOT n° BioT	43	49	52	52	55	51	303

The similarity level was calculated by using the Pearson product–moment correlation coefficient: isolates having 85% of similarity were grouped into the same biotype. The biodiversity index was calculated as ratio between number of biotypes and total number of isolates.

*At cheese factory C, the samples were collected at the end of September because in August the Trentingrana production stopped.

*Lactobacillus helveticus* was always dominant in NWS samples of all cheese factories, with the exception of cheese factory A. In particular, its relative presence ranged between 58 and 96% and was higher than 90% in NWS samples at cheese factories E and F. Conversely, *L. brevis* presence ranged between 0 and 42% with the exception of factory A where its relative presence was 51% and dominated NWS samples from May to November (Figure 1). *L. paracasei* and *L. plantarum* isolates were found only in NWS samples collected in February, and the higher presence was at cheese factories D and C, respectively. *L. fermentum* isolates were found only in NWS samples collected in May and more frequently at cheese factory D.

The distribution of the isolates into the NWS cultures was mainly related with the dairy plant rather than with the month of production. In fact, considering only the month of NWS production, there was no great difference in the distribution of *L. helveticus* and *L. brevis* isolates whose relative presence ranged between 62 and 89% and 11 and 34%, respectively.

### Characteristics of the Sequencing Data, Diversity Analysis, and Differential Abundance Analysis of the Bacterial Community in cNAW and NWS Samples

With the exception of two NWS samples, the extracted DNA was always successfully amplified in the bacterial V3–V4 16S rRNA gene region. A total of 2,082,145 paired-end sequences were obtained.

To address the hypothesis that species richness and biodiversity vary with sample source (cNAW or NWS sample), month (February, May, August, or November), and cheese factory (A, B, C, D, E, and F), the intragroup diversity estimation (*α* diversity) was calculated, using both the number of observed OTUs and Shannon diversity indexes. Both *α*-diversity indexes were not significantly different between cNAW and NWS samples (Kruskal–Wallis, *P* > 0.1), and no significant difference was found for month or dairy of sampling (data not shown).

**FIGURE 2:**
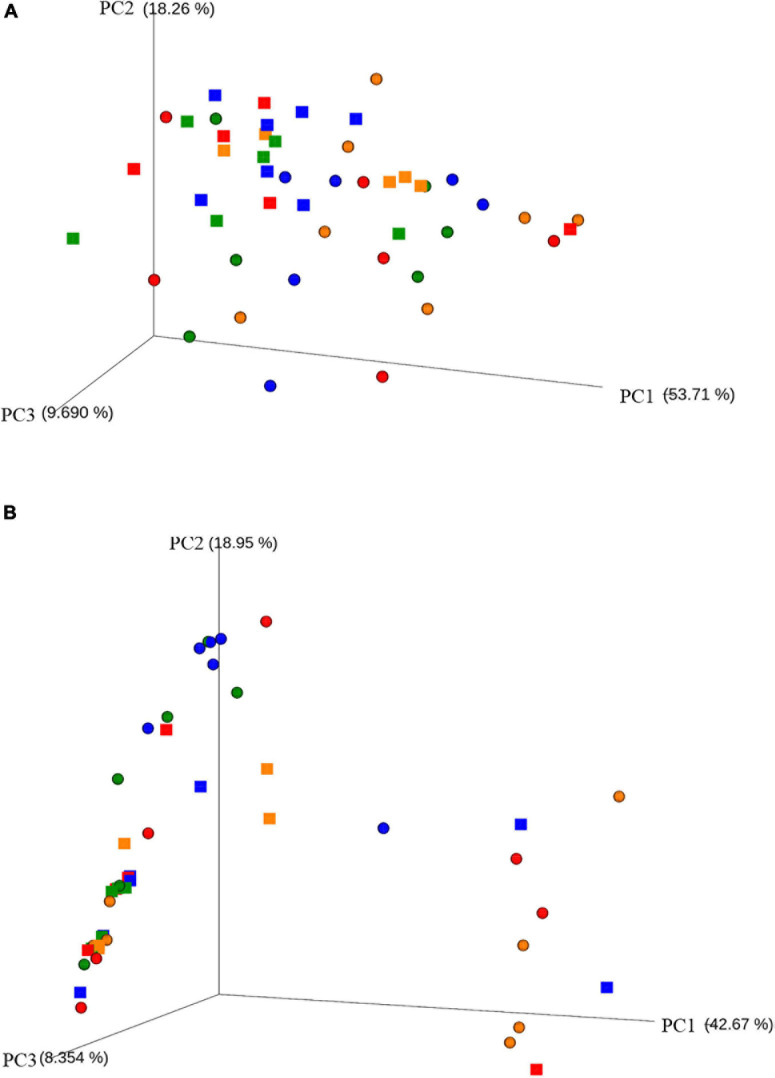
Principal coordinate analysis (PCoA) based on weighted **(A)** and unweighted **(B)** UniFrac distances of bacterial OTUs. **(A)** The first, second, and third axes explained 81.66% of the total variance. **(B)** The first, second, and third axes explained 69.97% of the total variance. Different symbols represent the different sampling sources: rings for cNAW and squares for NWS samples. Different colors represent the different sampling months: February = red, May = blue, August = orange and November = green.

In order to assess the amount of variation in bacteria composition among the samples, we calculated the phylogenetic *β* diversity based on both weighted and unweighted UniFrac distances. The PCoA plot onto weighted UniFrac distance matrix (Figure 2A) shows a little separation of the bacterial populations between cNAW (rings) and NWS samples (squares), visible on axis 2 explaining 18.3% of total variation. Conversely, the PCoA plot onto unweighted UniFrac distance matrix (Figure 2B) showed, with few exceptions, all cNAW and NWS samples overlapped in the same cloud on the left side of the graph. As the unweighted UniFrac distance accounts for the presence/absence of OTUs, so does the weighted UniFrac distance for abundance, although this result suggests that the individual microbial abundance more than the composition in species drives the distance among cNAW and NSW samples.

A PERMANOVA was performed to explore the effects and significance of the variables (Table 4): sample source (cNAW and NWS), cheese factory (A, B, C, D, E, and F), and month of sampling (February, May, August, and November). The test revealed that sample source affected microbial communities (*P* < 0.05); the month of sampling was slightly significant in cNAW and NSW samples, and in particular, the samples collected in November showed a significantly different bacterial community. Cheese factory was the main factor affecting microbial population diversity in all the samples, and the cheese factory D showed a significantly higher effect on cNAW and NSW samples (Table 4).

**TABLE 4:** PERMANOVA analysis (999 permutations) results for bacterial communities based on weighted UniFrac distances, respectively.

**Main effects**	**Pseudo-F**	***p* value**
Source of sample	4.25	0.012*
Month	1.553	0.111
Cheese factory	2.017	0.015*
**Pairwise comparisons for month**		
Feb vs. May	0.743	0.547
Feb vs. Aug	0.388	0.873
Feb vs. Nov	2.279	0.068*
May vs. Aug	1.430	0.211
May vs. Nov	2.083	0.065*
Aug vs. Nov	3.237	0.017*
**Pairwise comparisons for cheese factory**		
A vs. B	0.740	0.701
A vs. C	0.858	0.509
A vs. D	2.155	0.058*
A vs. E	2.983	0.169
A vs. F	2.006	0.128
B vs. C	1.200	0.276
B vs. D	2.112	0.044*
B vs. E	2.821	0.050*
B vs. F	2.259	0.090
C vs. D	3.089	0.006*
C vs. E	2.225	0.083
C vs. F	2.170	0.090
D vs. E	2.502	0.037*
D vs. F	1.504	0.066*
E vs. F	1.024	0.366

*The pairwise comparisons were calculated for different sampling sources collected from six dairy factories labeled from A to F four times a year from February to November 2018 (Feb = February, Aug = August, and Nov = November).*

Significance levels: *P < 0.07, **P < 0.01.

Considering only the OTUs whose relative abundance in each sample was greater than 0.01% (Table 5), *Firmicutes* was the dominant phylum, able to describe always more than 99% of the bacterial microbiota in all the samples. *L. helveticus* was always the dominant species over *Lactobacillus* spp. and *Streptococcus* spp., constituting more than 58% of the bacterial population in all the samples (Table 5). OTUs identified as *Chryseobacterium, Staphylococcus equorum, Lactococcus, Acinetobacter guillouiae*, and *Acinetobacter johnsonii* phylotypes were present in traces (<0.01%) and not in all the samples.

**TABLE 5:** Relative abundances of *L. helveticus*, *Lactobacillus*, and *Streptococcus* spp. of bacterial sequences from cNAW and NWS samples using Illumina MiSeq.

**Cheese factory**	**Sample**	***Lactobacillus* spp.**	***L. helveticus***	***Streptococcus* spp.**
A	cNAW	21.1 ± 13.9	75.3 ± 14.3	3.5 ± 1.3
	NWS	37.3 ± 6.7	60.5 ± 6.6	2.2 ± 0.3
B	cNAW	29.8 ± 4.4	62.6 ± 4.4	7.6 ± 1.0
	NWS	35.8 ± 5.7	61.7 ± 5.6	2.5 ± 0.9
C	cNAW	30.9 ± 11.6	68.1 ± 12.1	0.9 ± 0.8
	NWS	32.9 ± 10.9	65.1 ± 13.8	2.0 ± 1.3
D	cNAW	26.9 ± 3.6	71.7 ± 4.1	1.1 ± 0.8
	NWS	29.2 ± 8.2	69.8 ± 8.6	1.0 ± 0.5
E	cNAW	23.6 ± 8.5	74.2 ± 10.5	2.1 ± 1.9
	NWS	33.9 ± 10.0	64.9 ± 10.5	1.2 ± 0.6
F	cNAW	17.5 ± 6.2	80.1 ± 6.7	2.2 ± 1.0
	NWS	38.7 ± 6.7	58.6 ± 7.5	2.6 ± 1.0
**Month**				
February	cNAW	27.2 ± 13.4	69.1 ± 13.9	3.5 ± 2.2
	NWS	31.6 ± 5.4	66.6 ± 5.9	1.8 ± 1.1
May	cNAW	24.3 ± 6.8	72.4 ± 6.9	2.9 ± 2.7
	NWS	34.4 ± 5.0	64.1 ± 5.1	1.4 ± 1.1
August	cNAW	25.5 ± 9.6	75.4 ± 12.4	2.3 ± 3.5
	NWS	35.1 ± 4.9	62.1 ± 5.9	2.8 ± 1.8
November	cNAW	22.2 ± 8.8	75.4 ± 9.8	2.3 ± 2.6
	NWS	28.8 ± 8.1	70.2 ± 9.0	1.0 ± 0.9

*Samples were collected from six different cheese factories labeled from A to F for four months (February, May, August, and November). The data were expressed as means ± standard deviation of relative abundances transformed into a percentage.*

To identify taxonomic groups driving differences among the bacterial community, a differential abundance test by ANCOM method had been performed. No difference was found considering the month of sampling. Some taxa had been found differentially abundant among the cheese factories; in particular, both *Lactobacillus* and *Streptococcus* spp. were informative for characterizing the microbial community of cheese factories A and B.

### Dissemination and Titer of Phages in NWS Samples and Lysogenic State Analysis

All the 72 NWS samples collected from the six local cheese factories had been phage screened. The bacterial biotypes isolated from the different NWS cultures (Table 3) represented a reliable set of isolates suitable as hosts for phage detection in NWS samples; consequently, for each NWS sample screening, a set of putative bacterial hosts was selected, grouping all the isolates representative for each different biotype found in each NWS sample.

One hundred twenty phages were readily detected in 41 of the 72 NWS samples (Table 6) and were all lytic for their *L. helveticus* host isolate, with the exception of two phages lytic for *L. fermentum.* To detect the presence of prophage in their genomes, the 120 host bacterial isolates were treated with sublethal concentrations of MytC^+^. In none of the isolates, the MytC^+^ induced a sharp decrease in plate counts, confirming the lytic nature of all the phages.

**TABLE 6:** Distribution of phage isolates (n° Is.) from NWS samples collected from six dairy factories labeled from A to F, four times a year from February to November 2018.

	**A**	**B**	**C**	**D**	**E**	**F**	**TOT**
February	0	2 (1)	0	0	6 (6)	4 (1)	12 (8)
May	8 (2)	4 (0)	8 (1)	6 (3)	17 (8)	10 (5)	53 (19)
August	3 (2)	4 (1)	*3 (2)	3 (1)	4 (0)	12 (8)	29 (14)
November	3 (0)	1 (1)	1 (1)	5 (2)	2 (0)	14 (9)	29 (15)
Cheese Factory TOT	14 (4)	11 (3)	12 (4)	14 (6)	29 (14)	40 (23)	120 (54)
Presence (%) in the cheese factory	52%	26%	36%	30%	56%	85%	49%

*In the bracket, the number of phages able to form lysis plaques by agar layer technique on MRS-Ca.*

The presence in the cheese factory was calculated as ratio between number of isolated phages and number of tested bacterial host.

*At cheese factory C, the samples were collected at the end of September because in August the Trentingrana production stopped.

Phage titers were determined in all the 41 positive NWS samples. For *L. helveticus* phages, titers ranged between 2 × 10^6^ and 9 × 10^8^ pfu/mL with the exception of all the 12 phages from dairy factory C and six phages from dairy factory F, whose titers were in a range between 2 × 10^2^ and 4 × 10^5^ pfu/mL.

## Discussion

The NWS cultures used in Grana-like cheese production originate from artisanal back-slopping practices and face several selective pressures during the production process such as heat treatments and strong lactic acid fermentation. The bacterial consortia are characterized by a lower species complexity but high degree of strains diversity (Erkus et al., 2013), and bacteriophages have a regulatory role in the bacterial population dynamics through density-dependent predation (Rodriguez-Valera et al., 2009).

This study was carried out to investigate the bacterial and phage dynamics of cNAW and NWS production analyzing samples collected in 72 different days of Trentingrana production in six dairy plants, over a year period, by means of a combined approach, using plate counts, bacterial isolation, metataxonomic analysis, and phage isolation.

The NWS culture thermophilic counts, acidity, and pH were not significantly different for month (with exception of cheese factory C) or cheese factory, and their values were according to previously studied NWS cultures for Trentingrana (Franciosi et al., 2012) and Parmigiano Reggiano (Coppola et al., 2000). The homogeneity of the microbial counts throughout the overall sampling year could be the consequence of the consolidated process of NWS production from the cNAW, which guarantees for each dairy plant a microbial homogeneity of the NWS cultures. At cheese factory C, the thermophilic counts of NWS cultures were significantly higher in September and November. This cheese factory was the only one to stop the Trentingrana production in July and August. In September, the production of NWS cultures started again with the cheese. Therefore, this difference in thermophilic counts at dairy plant C could be a consequence of the total change in the NWS cultures that in September were no more linked to the NWS cultures of the previous months.

The sequencing of the 16S ribosomal gene of the isolates dominating the thermophilic microbial population in NWS cultures revealed the presence of five species, of which *L. helveticus* and *L. brevis* were the two dominant ones (95% of the thermophilic analyzed isolates). *L. paracasei*, *L. plantarum*, and *L. fermentum* were present in few NWS samples collected only in February and May. *L. helveticus* is well known as dominant in NWS cultures for Grana-like cheeses (Rossetti et al., 2008; Morandi et al., 2019; Bertani et al., 2020). *L. fermentum* had already been isolated in low abundance in NWS cultures of Grana-like cheeses (Rossetti et al., 2008; Morandi et al., 2019; Bertani et al., 2020) and the presence of two biotypes of *L. paracasei* and three of *L. plantarum* could be explained as an occasionally NWS culture contamination that occurred only in the February month of sampling. By contrast, the dominance of *L. brevis* together with *L. helveticus* in the thermophilic community of the NWS cultures in three cheese factories (*L. brevis* constituted > 30% of the isolates in A, B, and C) and during the overall year was surprising. The presence of 51 biotypes of *L. brevis* could not be explained as a contamination. *L. brevis* is one of the most common nonstarter LAB species found in the dairy industry (Settanni and Moschetti, 2010) and was already found in traces among the mesophilic bacteria of NWS cultures used for Parmigiano Reggiano (Coppola et al., 2000). To our knowledge, this is the first time this species had been isolated as one of the codominant thermophilic bacteria in NWS for Grana-like cheeses. *L. brevis* was in higher abundance in NWS from cheese factories A, B, and C where the overnight NWS production was dominated by a mesophilic stage with temperature of less than 40°C that is more selective for *L. brevis* than *L. helveticus*. As *L. brevis* growth could be strongly inhibited by the presence of homofermentative bacteria such as *L. helveticus* (Laleye et al., 1989), we speculate that a longer thermophilic stage greater than 40°C could be desirable because it is more selective for the growth of *L. helveticus* over *L. brevis* during the NWS culture production. *L. brevis* is an obligate heterofermentative bacteria producing CO_2_ from lactose and known to be a possible cause of early gas production in cheeses (Michael and Mullan, 2000); therefore, it is better to limit its abundance in NWS cultures.

Cluster analysis of RAPD-PCR bacterial isolates profiles showed a higher genotypic diversity within *L. helveticus* than *L. brevis* isolates; in fact, the 383 isolates of *L. helveticus* grouped into 243 biotypes, whereas the 120 *L. brevis* into 51 biotypes. The better adaptability of the *L. helveticus* species to the stressing conditions caused by the strongly acidic environment of the NWS culture may explain its higher strain diversity and consequently the lower *L. brevis* diversity at strain level. Many *L. helveticus* strains were probably able to adapt to acidity and high temperature of NWS cultures, and conversely, fewer *L. brevis* biotypes were resistant to these same stress conditions. The qualitative distribution of the biotypes into the different cultures was mainly dairy plant specific rather than correlated with the month of production. This is confirming our previous speculation—that the dominant LAB species of the NWS cultures are modulated by the variability in the parameters of the culture preparation that are different in each dairy plant. NWS cultures characterized by a low species-level complexity but a higher strain-level diversity were already described in other studies performed on dairy starter cultures strains (Frantzen et al., 2018; Schmid et al., 2018; Somerville et al., 2019). The differences in NWS culture production associated with the dairy plant characteristics (farmers, dairy factory operators, and environment) may affect the selection of NWS culture biotypes. Previous studies have also highlighted that NWS species and biotypes show different ability to adapt to dairy ecosystem (Giraffa and Neviani, 1999; Coppola et al., 2000; Moser et al., 2018).

Regarding the relative composition of the bacterial community, the *α* indices did not show significant differences in the biodiversity of the microbial communities of cNAW and NWS samples. The *β*-diversity analysis showed that the same bacterial species were characterizing both NWS and cNAW samples but with different relative abundances, and the dairy factory had a higher significant effect than the month of sampling on the abundance of the different species inside the microbial community. Dairy factory was already considered the most important source of variation of the Trentingrana cheese quality index (Bittante et al., 2011), and our results are in agreement with this previous study. The pairwise comparison showed that the species and relative abundances of cNAW and NWS cultures were very similar in all the dairy plants with the exception of the cheese factory D, whose NWS samples showed the highest relative abundance of *L. helveticus* and the lowest of *Lactobacillus* spp. and *Streptococcus thermophilus.* The high abundance of *L. helveticus* could be due to the NWS cycle of production: dairy plant D was the only cheese factory where the NWS cultures were produced with a thermophilic stage significantly longer than the mesophilic one (approximately 7 h longer), confirming the importance of NWS technology of production in modulating the microbial community of the NWS cultures. The analysis of both metataxonomic and quantitative data obtained by plate counts and isolation suggested that *L. helveticus* decreased in relative abundance but increased in amount from cNAW to NWS. This was in agreement with Bertani et al. (2020), showing that *L. helveticus* growth was mainly favored, in the acidic conditions occurring during the overnight fermentation of NWS. In Trentingrana dairy plants A, B, and C, the NWS cultures showed a high abundance of *L. brevis* isolates adapted to survive in thermophilic environment. Their dominance was also confirmed by metataxonomic data. *Lactobacillus* spp. relative abundance was very high (29–39%) when compared to previous metataxonomic studies on NWS for Grana-like cheeses (Bertani et al., 2020) where *Lactobacillus* spp. was never higher than 1%. *L. delbrueckii* was neither isolated nor sequenced in our study, and the abundance of *S. thermophilus* was never higher than 2.6% in NWS cultures. Both results were in agreement with a previous study by Morandi et al. (2019) on NWS cultures for Trentingrana production collected from 2014 to 2016, when a drastic reduction of *L. delbrueckii* and *S. thermophilus* isolates had been observed.

Ubiquitously distributed among all ecosystems, bacteriophages are commonly present in dairy plants (Pujato et al., 2019). It has been widely shown that all the most common starter bacteria species used in dairy industry, such as *S. thermophilus*, *Lactococcus*, and *Lactobacillus* spp. (Zago et al., 2006, 2015; Oliveira et al., 2018), are suitable hosts for phages, with consequent possible impairment in fermentations performance.

Almost all the 120 investigated phages were infecting the *L. helveticus* isolates, and 54 were able to form lysis plaque. This low ability in plaques forming was already observed in *L. helveticus* phages (Carminati et al., 2011; Zago et al., 2015). All the 120 phages were isolated over 1 year from NWS samples suggesting a simultaneous presence of phages and different sensitive bacteria within the cultures, as shown in previous studies (Zago et al., 2005, 2015). Although the widespread presence of lytic phages, all the NWS cultures were successful in their acidification activity, suggesting that the phages virulence could be counteracted by the presence of many bacteria, with different phage sensitivity, representing a natural way to control phage overwhelm. The individual resistance of the NWS isolates to phage predation could be one of the factors influencing NWS culture diversity as previously confirmed by Spus et al. (2015). In this study, the phage presence was not equally distributed among dairy plants; in fact, approximately 58% of the phages have been isolated from NWS cultures produced in only two dairy plants (E and F). It is remarkable that the same dairy plants E and F showed the lower biodiversity in the number of *L. helveticus* biotypes, confirming that the lower the bacterial biodiversity, the higher could be the phage presence. Few bacterial biotypes have been already observed in dairy starter cultures, where the phage predation operated mainly at strain rather than at species level (Erkus et al., 2013). The high biodiversity of dairy bacteria biotypes may prevent massive phage multiplication in NWS cultures and explain the recovery of a lower number of phages from the dairy plants with the higher biodiversity in *L. helveticus* biotypes. We speculate that, if phage activity changed in NWS culture, a population of few bacteria biotypes could be more susceptible to a phage predation with the consequent loss of NWS activity and generation of a defective cheese (Madera et al., 2004). Conversely, NWS cultures rich in different biotypes have a better chance to include phage-resistant bacteria able to counteract changes in phages community.

These considerations highlight the importance in assessing the strain biodiversity in NWS cultures: the higher the biodiversity, the higher the probability of a natural selection of bacterial strains resistant to phage predation and thus higher preservation of the overall activity of the NWS cultures, as also stated by Carminati et al. (2011).

In conclusion, this study added further knowledge on the microbial composition and diversity of NWS cultures for Grana-like cheeses in relation with the phage isolation. We speculate the importance of the technological process of NWS production and in particular of the thermophilic and mesophilic stages as drivers of the bacterial species dominant in NWS cultures. In addition, we found a possible correlation among bacterial starter biodiversity and the number of recovered lytic phages.

The abundance of phages in NWS cultures underlines again the importance of phage control strategies in the dairy industry. Ongoing studies are in progress to evaluate the roles of bacterial biodiversity, phages, and NWS production technology on the species composition of Trentingrana NWS cultures.

## Data Availability Statement

The datasets presented in this study can be found in online repositories. The names of the repository/repositories and accession number(s) can be found below: https://www.ncbi.nlm.nih.gov/, PRJNA695135.

## Author Contributions

EF and AMe devised the study. EF and AMa drafted the manuscript. EF, AMa, NC, AG, IC, and MR performed sampling plane and the experiments. MZ contributed to the study with her know-how on phages. EF, AMe, and KT provided resources and intellectual input that supported the study. All authors contributed to the article and approved the submitted version.

## Conflict of Interest

The authors declare that the research was conducted in the absence of any commercial or financial relationships that could be construed as a potential conflict of interest.

## Footnotes

1 https://github.com/yhwu/idemp/blob/master/idemp.cpp

2 https://github.com/qiime2/q2-feature-classifier

3 https://www.ncbi.nlm.nih.gov/bioproject/695135

4 www.statsoft.com
